# Supplementary material for: Micro- and nanoplastics alter electrophysiological brain patterns and reshape human neurodevelopmental trajectories
Source: bioRxiv. 2026 Sep 24:2026.09.18.752710. Preprint. [Version 1] doi: 10.64898/2026.09.18.752710 (PMC13622068; doi:10.64898/2026.09.18.752710)
Supplement: Supplementary file 1 [file NIHPP2026.09.18.752710v1-supplement-1.pdf]

bioRxiv preprint doi: https://doi.org/10.64898/2026.09.18.752710; this version posted September 24, 2026. The copyright holder for this preprint (which was not certified by peer review) is the author/funder, who has granted bioRxiv a license to display the preprint in perpetuity. It is made available under a CC-BY 4.0 International license.

Supplementary Figure Legends

Supplementary figure 1: Synthetic MNP characterization, experimental design, and longitudinal cortical organoid growth. (**a**) Brightfield images taken in the 40X objective for each sMNP used for CO exposure: polystyrene (PS), polyethylene terephthalate (PET), low-density polyethylene (LDPE), and high-density polyethylene (HDPE). (**b**) FTIR confirmation of each sMNP composition. Plastic composition was confirmed using ATR-FTIR. Spectra were compared to known peaks for respective polymers to confirm microplastic structure. From left to right: FTIR spectra for PS, PET, LDPE, HDPE. (**c**) Zeta potential (mV) of PS, PET, LDPE, and HDPE microplastic suspensions measured by electrophoretic light scattering using a Malvern ZetaSizer Ultra. Microplastics were dispersed in MilliQ water containing 0.05% Tween 20 and sonicated prior to analysis. Values are presented as mean ± SD. (**d**) Schematic overview of experimental design. Human iPSC-derived COs were exposed to sMNPs (10 or 1,000 µg ml^{-1} in PBS) beginning on day 1, with sMNPs replenished every 7 days. COs were cultured in differentiation media supplemented as indicated throughout the exposure period. Samples were collected after 8 d.p.e. or 30 d.p.e. for downstream analysis. Created with BioRender. (**e**) Size quantification of untreated, 10 µg ml^{-1} and 1,000 µg ml^{-1} sMNP-treated COs over 30 days of exposure. Data are presented as mean ± SD. n = 5-19 organoids per treatment group from a single organoid differentiation. (**f**) Representative fluorescence images of DAPI-stained untreated, 10 µg ml^{-1} and 1,000 µg ml^{-1} sMNP-treated COs, exposed on day 6 of growth and collected 30 d.p.e. The boxed region in the 1,000 µg ml^{-1} condition is shown at higher magnification to highlight particle aggregates. Scale bars = 200 µm (lower magnification) and 50 µm (higher magnification). (**g**) Heatmap of selected senescence associated genes identified from the SenSig[13] and SenMayo[14] gene sets in untreated and 1,000 µg ml^{-1} sMNP-treated COs, exposed on day 6 of growth and collected 8 d.p.e. Gene expression is displayed as row-wise Z-scores of normalized RNA-sequencing data, with orange representing higher relative expression and blue representing lower relative expression. (**h**) Heatmap of selected neuronal development and choroid plexus marker genes in untreated and 1,000 µg ml^{-1} sMNP treated COs, exposed on day 6 of growth and collected 8 d.p.e. Expression values are shown as row-wise Z-scores of normalized RNA-sequencing data, with orange representing higher

bioRxiv preprint doi: https://doi.org/10.64898/2026.09.18.752710; this version posted September 24, 2026. The copyright holder for this preprint (which was not certified by peer review) is the author/funder, who has granted bioRxiv a license to display the preprint in perpetuity. It is made available under a CC-BY 4.0 International license.

relative expression and blue representing lower relative expression. Genes are grouped according to their annotated biological function.

Supplementary figure 2: Low-resolution cell-type projection of sMNP-associated transcriptional signatures in cortical organoids. (**a**) t-SNE visualization of the whole body reference showing ten cell classes, including five brain-derived populations (excitatory neurons, interneurons, astrocytes, oligodendrocytes, and OPCs) and five broader whole-body classes (immune, epithelial, endothelial, stromal, and germline). Brain-derived populations were subsequently pooled into a single neural class for DEG projection analyses. (**b**) PCA visualization of single-cell reference populations based on the top 50 upregulated DEGs in cortical organoids exposed to sMNPs on day 6 of growth and collected 30 d.p.e., ranked by adjusted *P* value. Reference cells were derived from a merged Tabula Sapiens and Allen Brain Map dataset and colored according to cell class[15,16]. PCA plots display a class-balanced, stratified subsample of reference cells for visualization. **c,d,** Cell-type composition across PCA coordinate bins for PC1 (**c**) and PC2 (**e**) of the upregulated DEG signature in COs exposed to sMNPs on day 6 of growth and collected 30 d.p.e. Stacked bars represent the percentage of reference cells assigned to endothelial, epithelial, germline, immune, neural, and stromal cell classes within each PCA coordinate bin. (**e**) PCA visualization of single cell reference populations based on the top 50 downregulated DEGs in cortical organoids exposed to sMNPs on day 6 of growth and collected 30 d.p.e., ranked by adjusted *P* value. Reference cells were derived from a merged Tabula Sapiens and Allen Brain Map dataset and colored according to cell class[15,16]. PCA plots display a class-balanced, stratified subsample of reference cells for visualization. **f,g,** Cell-type composition across PCA coordinate bins for PC1 (**f**) and PC2 (**g**) of the downregulated DEG signature in COs exposed to sMNPs on day 6 of growth and collected 30 d.p.e. Stacked bars represent the percentage of reference cells assigned to endothelial, epithelial, germline, immune, neural, and stromal cell classes within each PCA coordinate bin.

Supplementary figure 3: High-resolution cell-type projection of sMNP-associated transcriptional signatures in cortical organoids. (**a**) t-SNE visualization of the whole-body reference at fine resolution, comprising 56 cell-type categories from the Allen Brain Map and Tabula Sapiens references[15,16]. Brain-derived populations remain individually

bioRxiv preprint doi: https://doi.org/10.64898/2026.09.18.752710; this version posted September 24, 2026. The copyright holder for this preprint (which was not certified by peer review) is the author/funder, who has granted bioRxiv a license to display the preprint in perpetuity. It is made available under a CC-BY 4.0 International license.

annotated at this stage. The color codes apply for the entire figure. (**b**) PCA visualization of reference cells based on the top 50 upregulated DEGs in cortical organoids exposed to sMNPs on day 6 of growth and collected 30 d.p.e., ranked by adjusted *P* value. **c,d,** Cell-type composition across PCA coordinate bins for PC1 (**c**) and PC2 (**d**) of the upregulated DEG signature in COs exposed to sMNPs on day 6 of growth and collected 30 d.p.e. Stacked bars represent the percentage of reference cells assigned to each fine-resolution cell type within each PCA coordinate bin. Selected cell-type groups are labeled within the composition plots to facilitate visualization of the predominant populations. PCA projections and cell-type compositions were generated using a stratified subsample for visualization. (**e**) PCA visualization of reference cells based on the top 50 downregulated DEG signatures in cortical organoids exposed to sMNPs on day 6 of growth and collected 30 d.p.e., ranked by adjusted *P* value. **f,g,** Cell-type composition across PCA coordinate bins for PC1 (**f**) and PC2 (**g**) of the downregulated DEG signature. Stacked bars represent the percentage of reference cells assigned to each fine-resolution cell type within each PCA coordinate bin. Selected cell-type groups are labeled within the composition plots to facilitate visualization of the predominant populations. PCA projections and cell-type compositions were generated using a stratified subsample for visualization.

Supplementary figure 4: sMNP exposure alters senescence-associated and neurodevelopmental transcriptional signatures in cortical organoids. a,b, Representative immunofluorescence images of untreated and 1,000 µg ml^{-1} sMNP treated COs, exposed on day 6 of growth and collected 8 d.p.e. (**a**) and 30 d.p.e. (**b**). Sections were stained for PAX6 (green), and p16 (yellow). Nuclei were counterstained with DAPI (blue). Merged images are shown for each staining panel. Scale bars = 200 µm for 8 d.p.e. images and 250 µm for 30 d.p.e. images. **c,d,** Quantification of PAX6 positive and p16 positive cells in untreated and 1,000 µg ml^{-1} sMNP treated COs, exposed on day 6 of growth and collected 8 d.p.e. (**c**) and 30 d.p.e. (**d**). Data are presented as mean ± SD, with each dot representing an individual organoid. Statistical significance was determined using a two-tailed Welch's *t*-test. *n*=3 organoids per group. Exact *P* values are shown.

bioRxiv preprint doi: https://doi.org/10.64898/2026.09.18.752710; this version posted September 24, 2026. The copyright holder for this preprint (which was not certified by peer review) is the author/funder, who has granted bioRxiv a license to display the preprint in perpetuity. It is made available under a CC-BY 4.0 International license.

Supplementary figure 5: Experimental design and characterization of cortical organoid growth following polymer-specific microplastic exposure. (**a**) Schematic overview of experimental design. Human iPSC-derived COs were treated with individual polymers at 1,000 µg ml^{-1} in PBS, beginning on day 1, with polymers replenished every 7 days. COs were cultured in differentiation media supplemented as indicated throughout the exposure period. Samples were collected after 8 d.p.e. and 30 d.p.e. for downstream analysis. Created with BioRender. (**b**) Size quantification of untreated and 1,000 µg ml^{-1} PS, PET, LDPE, or HDPE-treated COs over 8 days of exposure. Data are presented as mean ± SD. Significance was determined by *p*-value with ***=p<0.0001, *=p<0.05. n = 6-14 organoids per treatment group from a single organoid differentiation. **c,d,** Brightfield images of COs exposed to PS, PET, LDPE or HDPE on day 6 of growth and collected 8 d.p.e. (**c**) and 30 d.p.e. (**d**). Low-magnification images (left) show overall organoid morphology, while higher-magnification images (right) highlight the organoid surface and the presence of associated sMNP particles, indicated by red arrows. Images are representative of independent organoids from each treatment group. Scale bars = 200 µm for lower magnification and 50 µm for higher magnification.

Supplementary figure 6: Polymer-specific effects on neural progenitor, neuronal, and senescence-associated markers in cortical organoids. a,c,e, Representative immunofluorescence images of untreated and 1,000 µg ml^{-1} PS, PET, LDPE, or HDPE-treated COs, exposed on day 6 of growth and collected 30 d.p.e. Sections were stained for SOX2 (green) and p21 (yellow) (**a**); MAP2 (yellow) (**c**); PAX6 (green) and p16 (yellow) (**e**). Nuclei were counterstained with DAPI (blue). Merged images are shown for each treatment group. Scale bars = 400 µm for untreated, PS, and LDPE SOX2/p21, all MAP2 images and all PAX6/p16 images; 250 µm for PET and HDPE SOX2/p21 images. **b,d,f,** Quantification of SOX2-positive and p21-positive (**b**); MAP2-positive (**d**); PAX6 positive and p16 positive (**f**) cells in untreated and 1,000 µg ml^{-1} PS, PET, LDPE, or HDPE-treated COs, exposed on day 6 of growth and collected 30 d.p.e. Data are presented as mean ± SD, with each data point representing an individual organoid. Data shown are batch-corrected to account for variability between differentiations. Statistical significance was determined using a one-way ANOVA followed by Dunnett's multiple comparisons test. Exact *P* values are shown. n=4-14 organoids per treatment group.

bioRxiv preprint doi: https://doi.org/10.64898/2026.09.18.752710; this version posted September 24, 2026. The copyright holder for this preprint (which was not certified by peer review) is the author/funder, who has granted bioRxiv a license to display the preprint in perpetuity. It is made available under a CC-BY 4.0 International license.

Supplementary figure 7: Individual sMNP polymers differentially alter neural progenitor identity, cellular senescence, and tight-junction organization in developing COs. a,c, Representative immunofluorescence images of untreated and 1,000 μg ml^{-1} PS, PET, LDPE, or HDPE-treated COs, exposed on day 6 of growth and collected 8 d.p.e. Sections were stained for SOX2 (green) and p21 (yellow) (**a**); ZO-1 (green) and PAX6 (yellow) (**c**). Nuclei were counterstained with DAPI (blue). Merged images are shown for each treatment group. Scale bars = 200 μm. **b,d,** Quantification of SOX2 positive and p21 positive (**b**); ZO-1 positive and PAX6 positive (**d**) cells in untreated and 1,000 μg ml^{-1} PS, PET, LDPE, or HDPE-treated COs exposed on day 6 of growth and collected 8 d.p.e. Data are presented as mean ± SD, with each data point representing an individual organoid. Statistical significance was determined using a one-way ANOVA followed by Dunnett's multiple comparisons test. Exact *P* values are shown. n = 5-7 organoids per treatment group from a single organoid differentiation.

Supplementary figure 8: Generation, polymer composition, and transcriptional profiling of environmentally derived micro- and nanoplastics (eMNPs). (**a**) Schematic overview of eMNP generation from plastic debris collected at Kamilo Beach, Hawaii. Plastic debris was manually size-reduced, washed with soap and water followed by 100% ethanol, cryogenically milled for three cycles, and subjected to 5 weeks of UV and ozone weathering. The weathered material was subsequently re-milled and size-fractionated to generate particles of defined size ranges, including 500, 250, 125, 63, and <20 μm, before exposing organoids on day 6 of growth. (**b**) Polymer composition of the resulting eMNP preparation, consisting primarily of polyethylene (PE) and polypropylene (PP), with additional contributions from polyurethane (PU), nylon 66 (N66), polymethyl methacrylate (PMMA), polyvinyl chloride (PVC), polyethylene terephthalate (PET), nylon 6 (N6), acrylonitrile butadiene styrene (ABS), styrene-butadiene rubber (SBR), polycarbonate (PC), and polystyrene (PS). (**c**) UMAP visualization of merged transcriptional clusters across untreated, and 1,000 μg ml^{-1} sMNP or eMNP -treated COs exposed on day 6 of growth and collected 30 d.p.e. Each panel displays nuclei colored by cluster identity, with the same UMAP embedding and cluster assignments shown across conditions. Cluster numbers correspond to the merged cell-state annotations described in the text. Untreated, *n* = 8,824 nuclei; sMNP, *n* = 11,067 nuclei; eMNP, *n* =

bioRxiv preprint doi: https://doi.org/10.64898/2026.09.18.752710; this version posted September 24, 2026. The copyright holder for this preprint (which was not certified by peer review) is the author/funder, who has granted bioRxiv a license to display the preprint in perpetuity. It is made available under a CC-BY 4.0 International license.

12,215 nuclei. Gray points represent nuclei from all three conditions and provide the reference distribution for each condition-specific UMAP.

Supplementary figure 9: Cellular characterization of cortical organoids following sMNP and eMNP exposure. a,c,e,g, Representative immunofluorescence images of untreated, 1,000 µg ml^{-1} sMNP, or eMNP -treated COs exposed on day 6 of growth and collected 30 d.p.e. Sections were stained for ZO-1 (green), and TTR (yellow) (**a**); NeuN (green), and p21 (yellow) (**c**); SOX2 (green), and MAP2 (yellow) (**e**); GFAP (green), and p16 (yellow) (**g**). Nuclei were counterstained with DAPI (blue). Merged images are shown for each staining panel. Scale bars = 200 µm for all images. **b,d,f,h,** Quantification of ZO-1-positive and TTR-positive (**b**); NeuN-positive and p21-positive (**d**); SOX2-positive and MAP2-positive (**f**); GFAP-positive, and p16-positive (**h**) cells in untreated, 1,000 µg ml^{-1} sMNP, or eMNP-treated COs exposed on day 6 of growth and collected 30 d.p.e. Data are presented as mean ± SD, with each data point representing an individual organoid. Statistical significance was determined using a one-way ANOVA followed by Dunnett's multiple comparisons test. Exact *P* values are shown. n= 5-9 organoids per treatment group from a single organoid differentiation.

Supplementary figure 10: MNP exposure alters cellular phenotypes in mature ventral midbrain organoids. a,c,e, Representative immunofluorescence images of untreated, 1,000 µg ml^{-1} sMNP, or eMNP-treated VMOs, exposed on day 90 of growth and collected 30 d.p.e. Sections were stained for LMX1A (green), and p21 (yellow) (**a**); TTR (green), and SOX2 (yellow) (**c**); and TH (yellow) (**e**). Nuclei were counterstained with DAPI (blue). Merged images are shown for each staining panel. Scale bars = 200 µm for GFAP/TH images; 400 µm for untreated and 250 µm for sMNP- and eMNP-treated LMX1A/p21 images; and 400 µm for untreated and eMNP-treated TTR/SOX2 images and 250 µm for sMNPs-treated TTR/SOX2 images. **b,d,f,** Quantification of LMX1A-positive and p21-positive (**b**); TTR-positive, and SOX2-positive (**d**); and TH-positive (**f**) cells in untreated, 1,000 µg ml^{-1} sMNP, or eMNP-treated VMOs, exposed on day 90 of growth and collected 30 d.p.e. Data are presented as mean ± SD, with each data point representing an individual organoid. Statistical significance was determined using a one-way ANOVA followed by Dunnett's multiple comparisons test. Exact *P* values are shown. n=3-7 organoids per treatment group from a single organoid differentiation.

bioRxiv preprint doi: https://doi.org/10.64898/2026.09.18.752710; this version posted September 24, 2026. The copyright holder for this preprint (which was not certified by peer review) is the author/funder, who has granted bioRxiv a license to display the preprint in perpetuity. It is made available under a CC-BY 4.0 International license.

Supplementary figure 11: Functional activity and network organization of MNP-exposed ventral midbrain organoids. (**a**) Representative functional networks from an untreated organoid, an sMNP-exposed organoid, and an eMNP-exposed organoid. Each circle represents one of the displayed electrodes, and the connecting lines indicate functional relationships among electrodes based on coordinated spike timing, as measured using the spike-time tiling coefficient (STTC). The panel displays the top 20% of connected electrodes by node degree and up to 40 of their strongest mutual connections. Larger circles indicate electrodes with more functional connections, while thicker lines indicate stronger STTC connectivity. Node color represents the participation coefficient. Values closer to zero indicate predominantly within-module connectivity, whereas higher values indicate connections distributed across multiple modules. The small insets show where the displayed electrodes are located on the complete electrode array.

Supplementary Figure 1

a

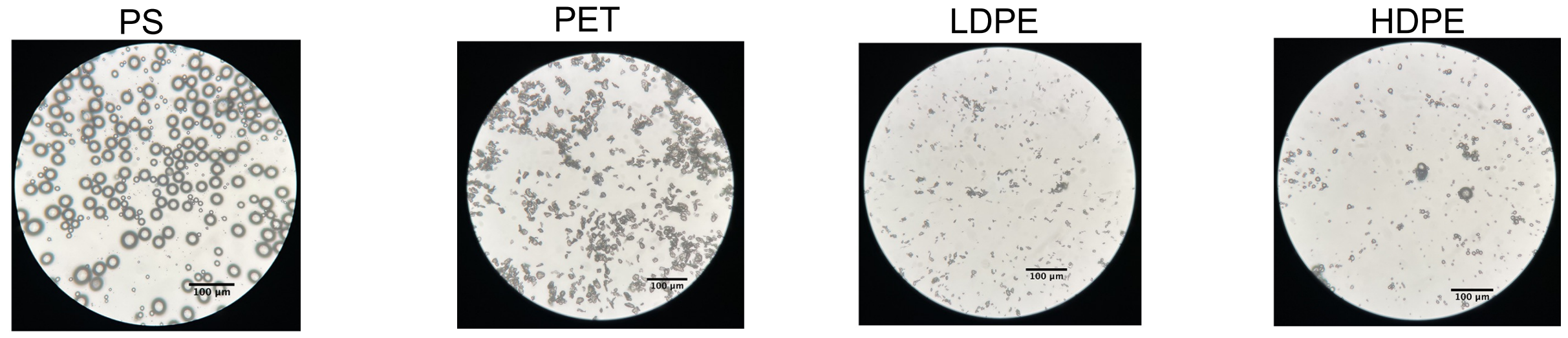

b

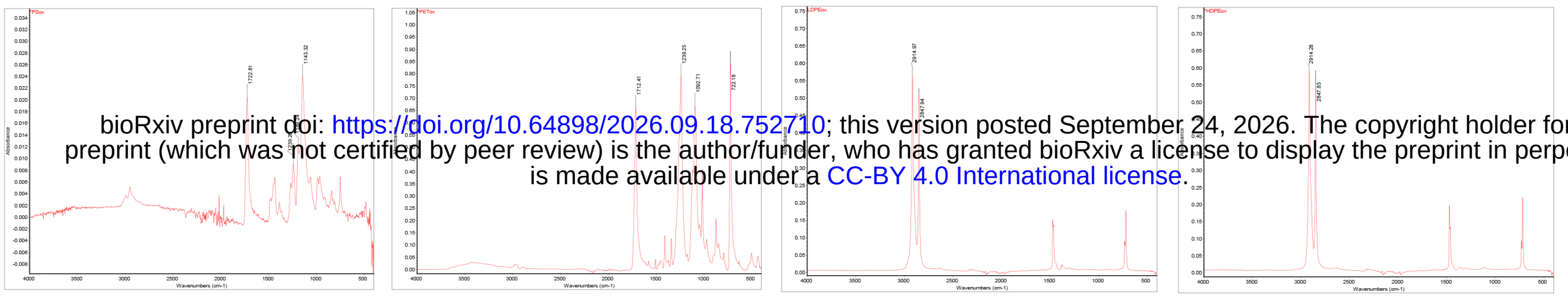

bioRxiv preprint doi: https://doi.org/10.64898/2026.09.18.752710; this version posted September 24, 2026. The copyright holder for this preprint (which was not certified by peer review) is the author/funder, who has granted bioRxiv a license to display the preprint in perpetuity. It is made available under a CC-BY 4.0 International license.

c

Plastic Type	Zeta Potential (mV)	Standard Deviation
**PS**	-15.07	3.15
**PET**	-12.11	1.22
**LDPE**	-27.46	1.51
**HDPE**	-13.82	0.07

d

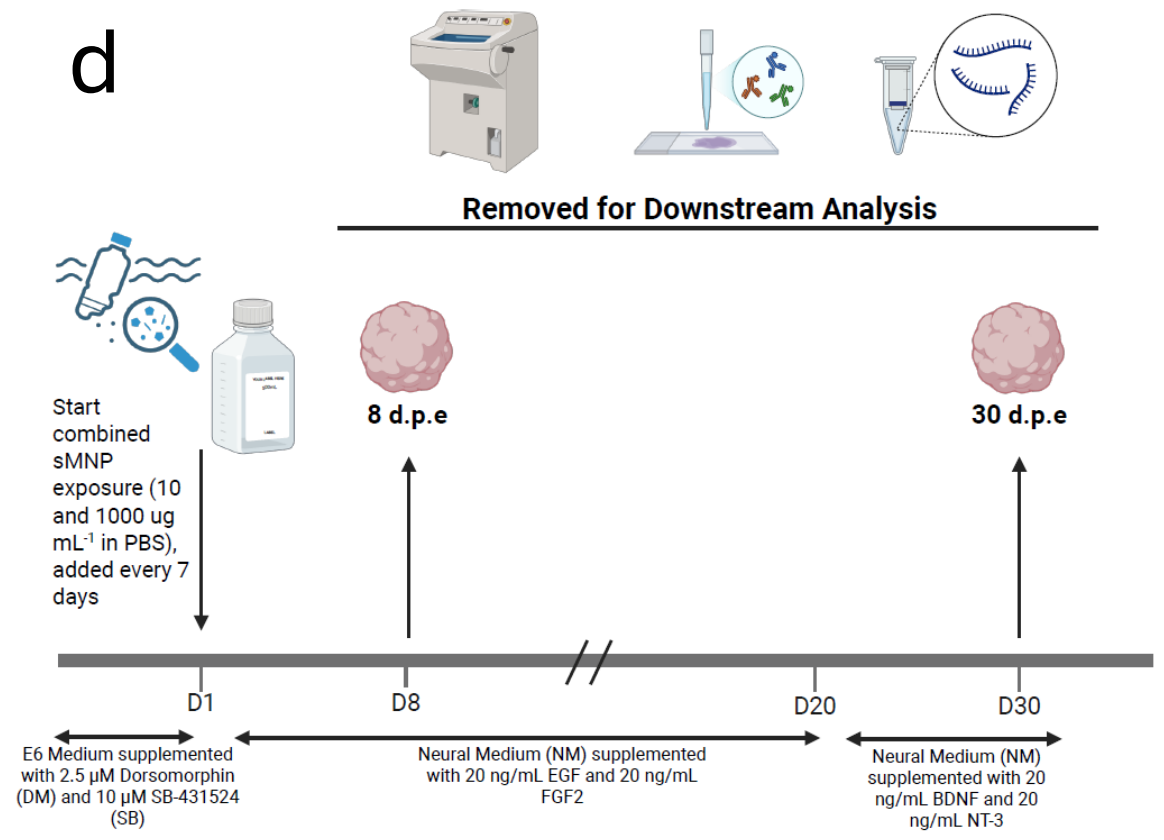

e

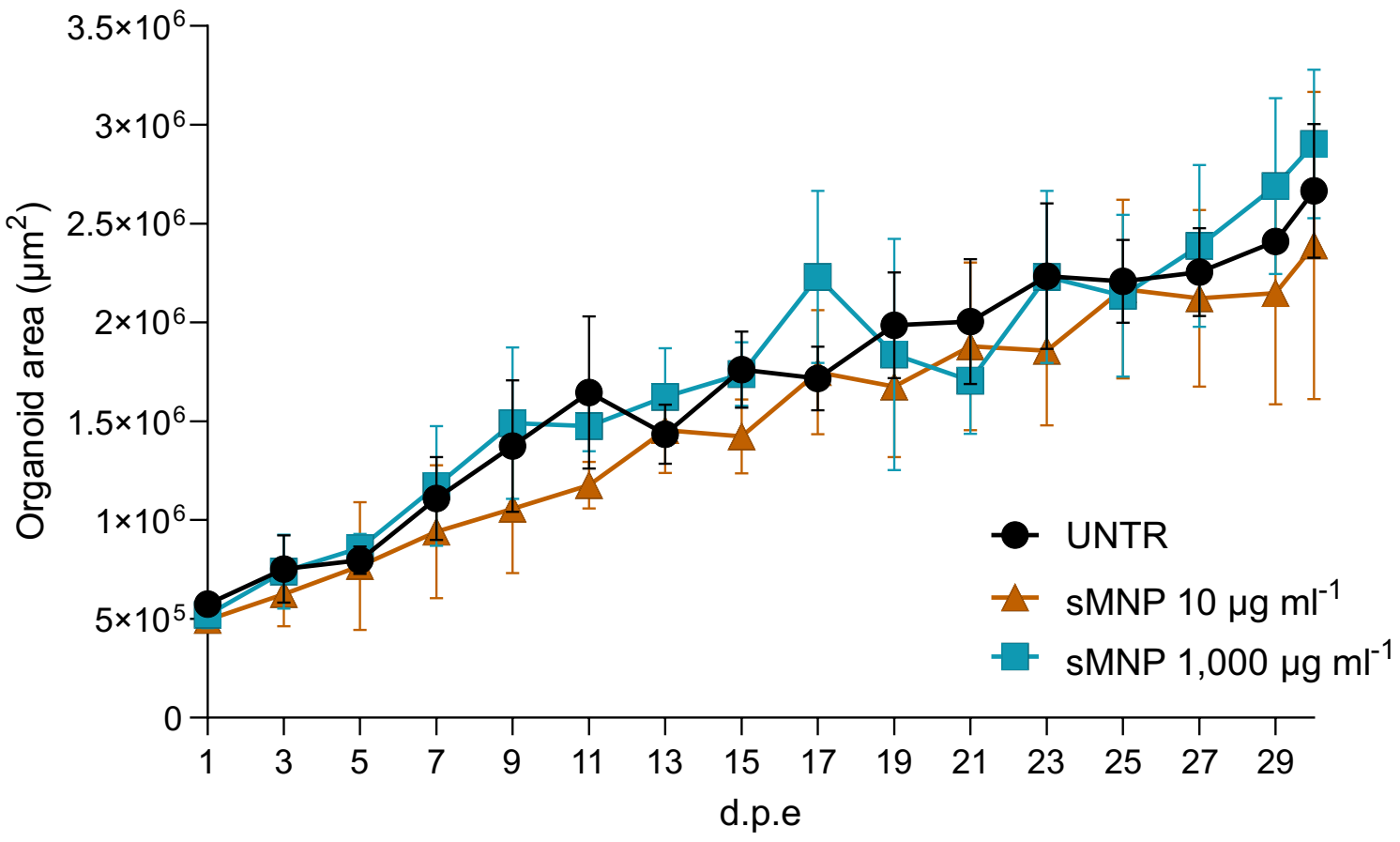

f

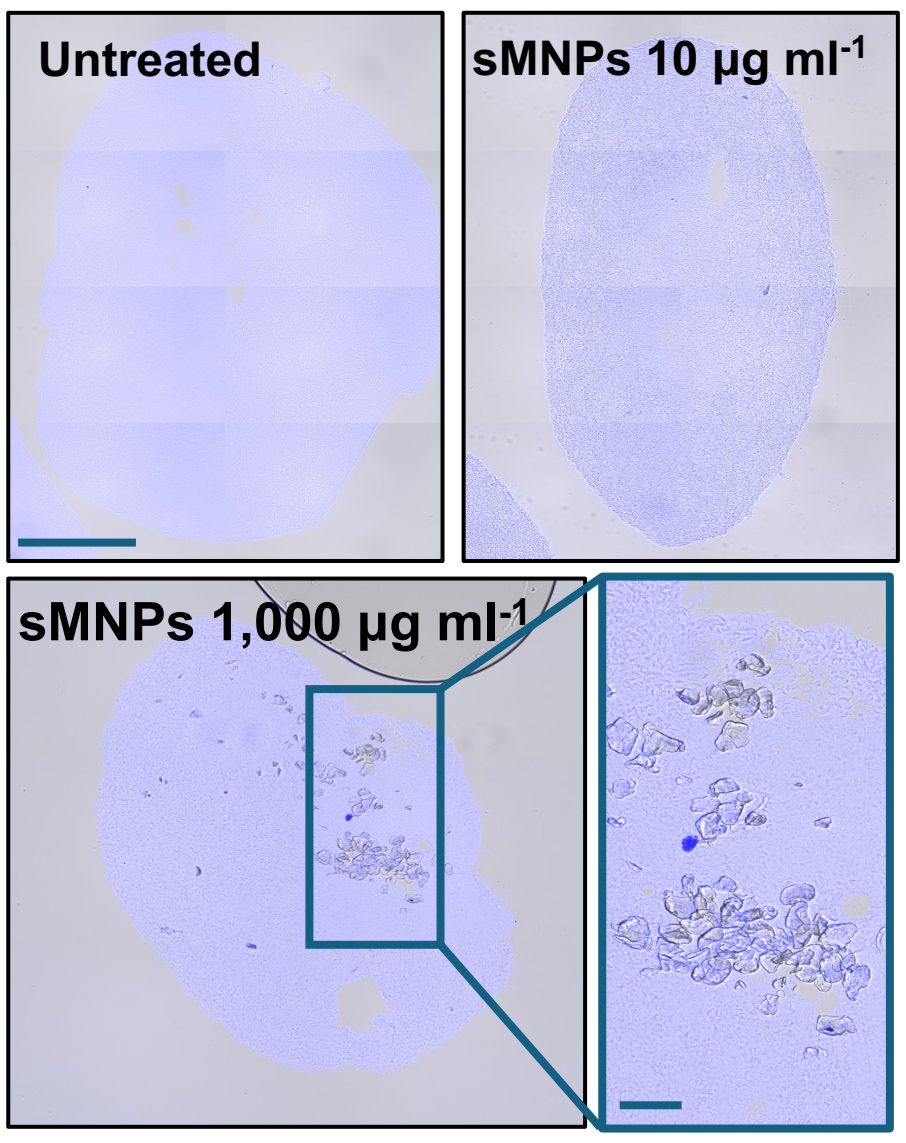

g

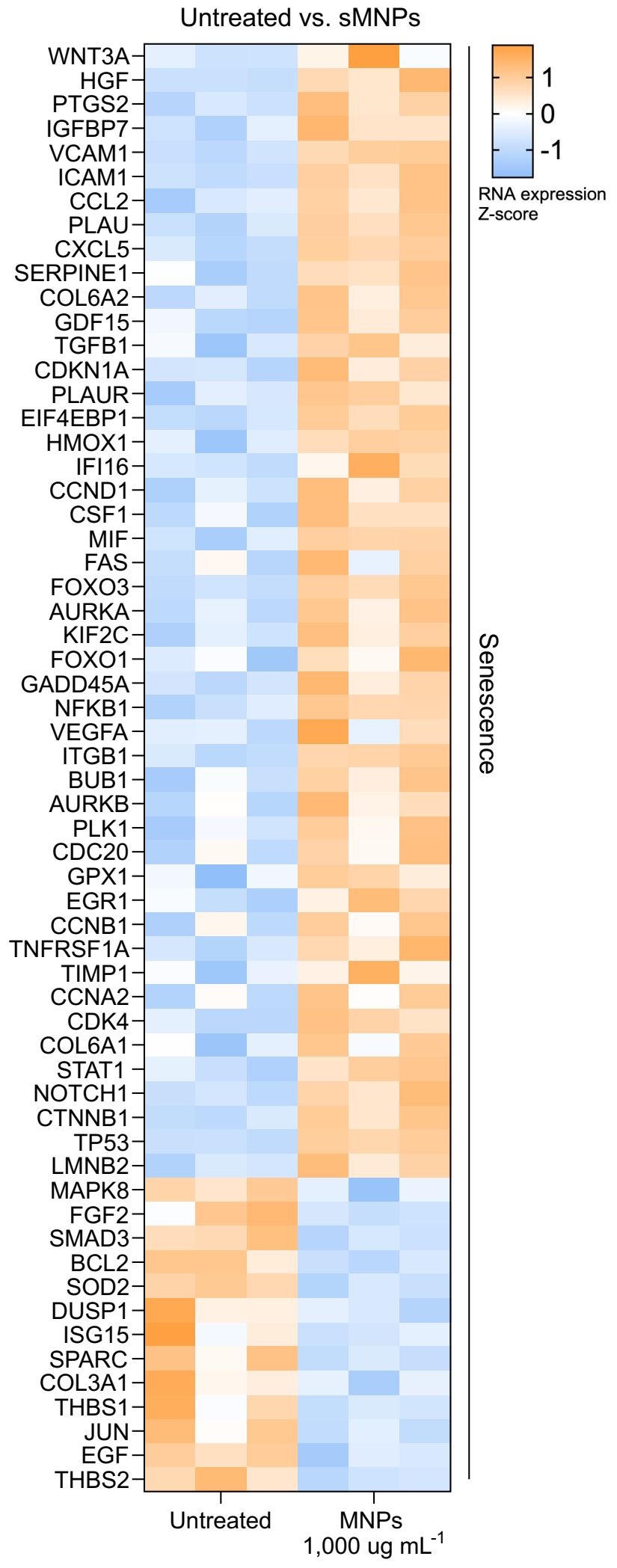

h

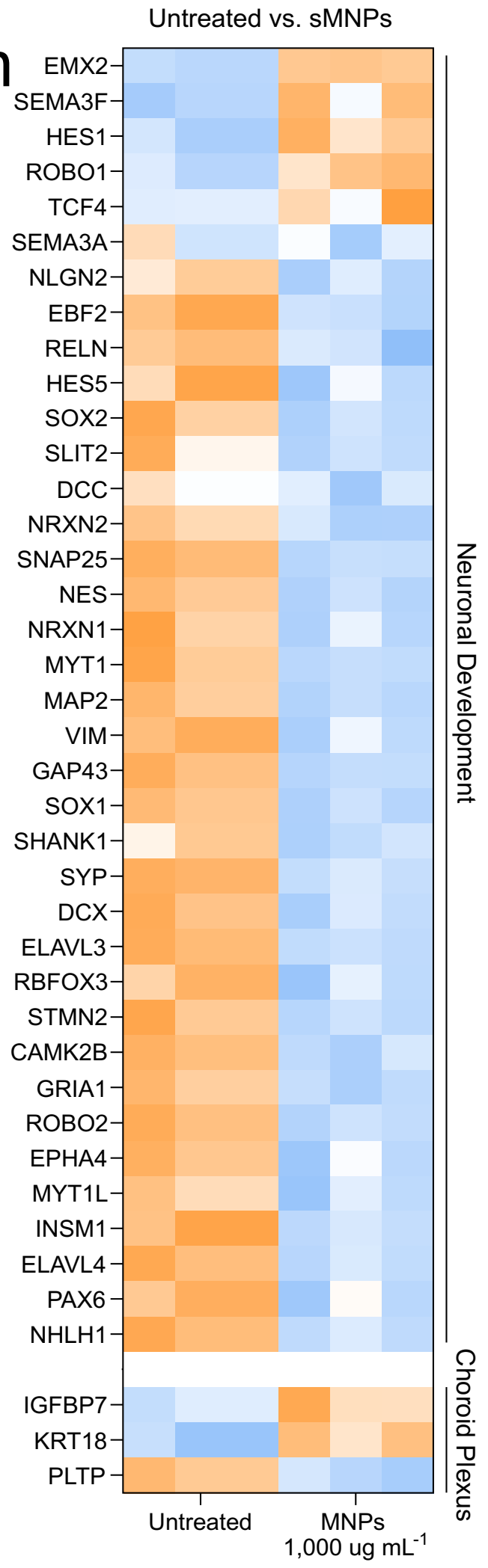

Supplementary Figure 2

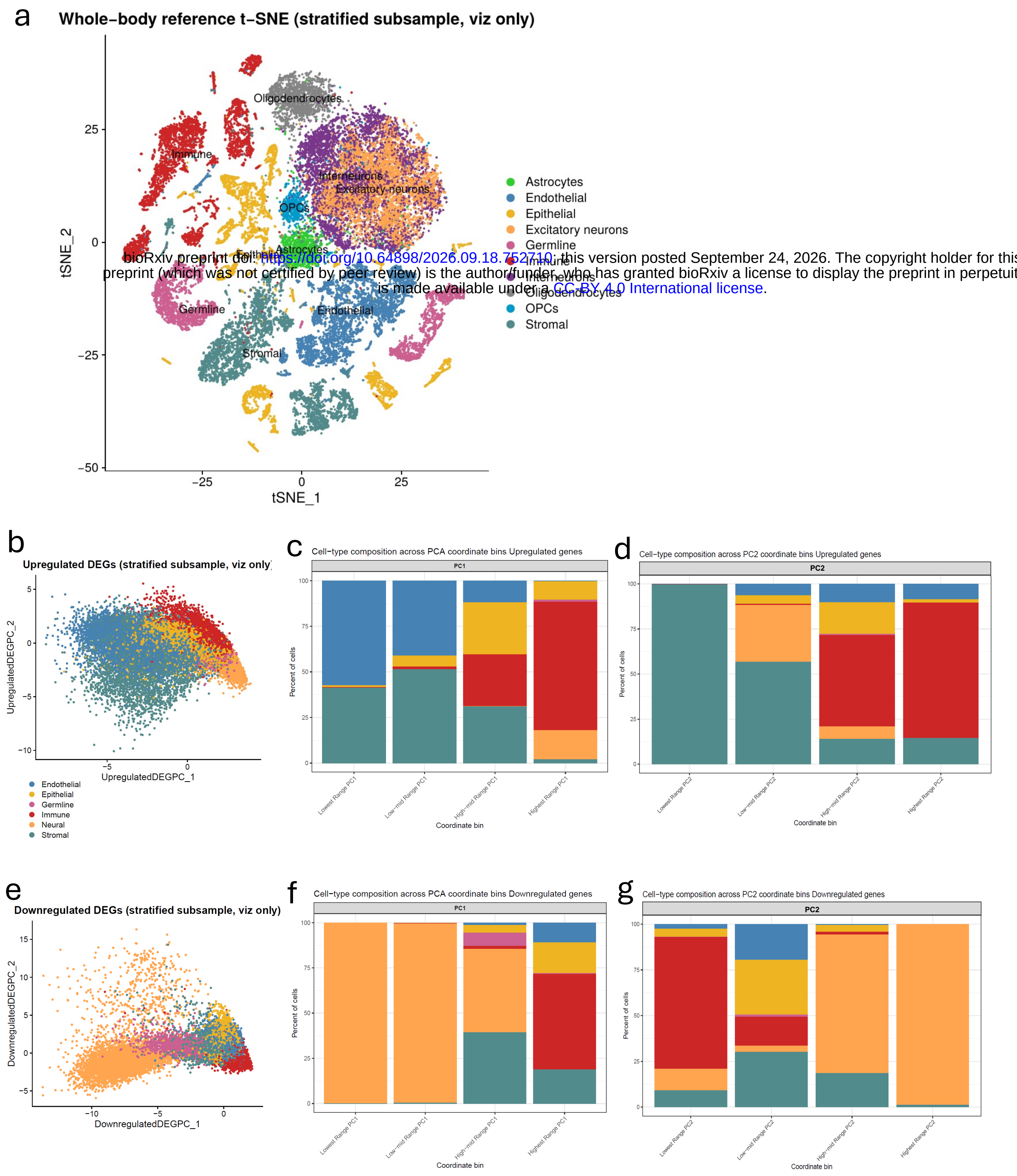

bioRxiv preprint doi: https://doi.org/10.64898/2026.09.18.752710; this version posted September 24, 2026. The copyright holder for this preprint (which was not certified by peer review) is the author/funder, who has granted bioRxiv a license to display the preprint in perpetuity. It is made available under a CC-BY 4.0 International license.

Supplementary Figure 3

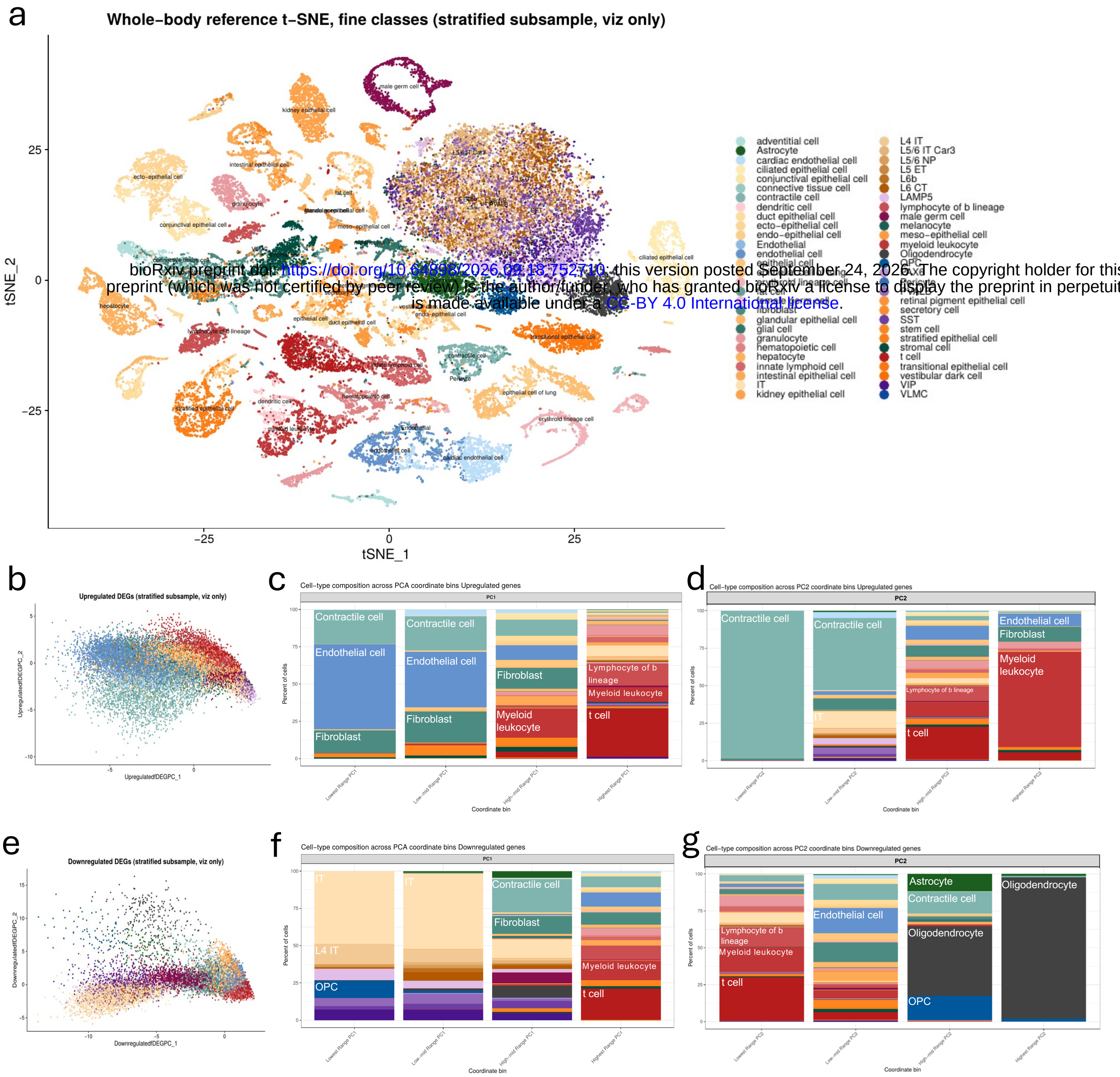

bioRxiv preprint doi: https://doi.org/10.64898/2026.09.18.752710; this version posted September 24, 2026. The copyright holder for this preprint (which was not certified by peer review) is the author/funder, who has granted bioRxiv a license to display the preprint in perpetuit
is made available under a CC-BY 4.0 International license.

Supplementary Figure 4

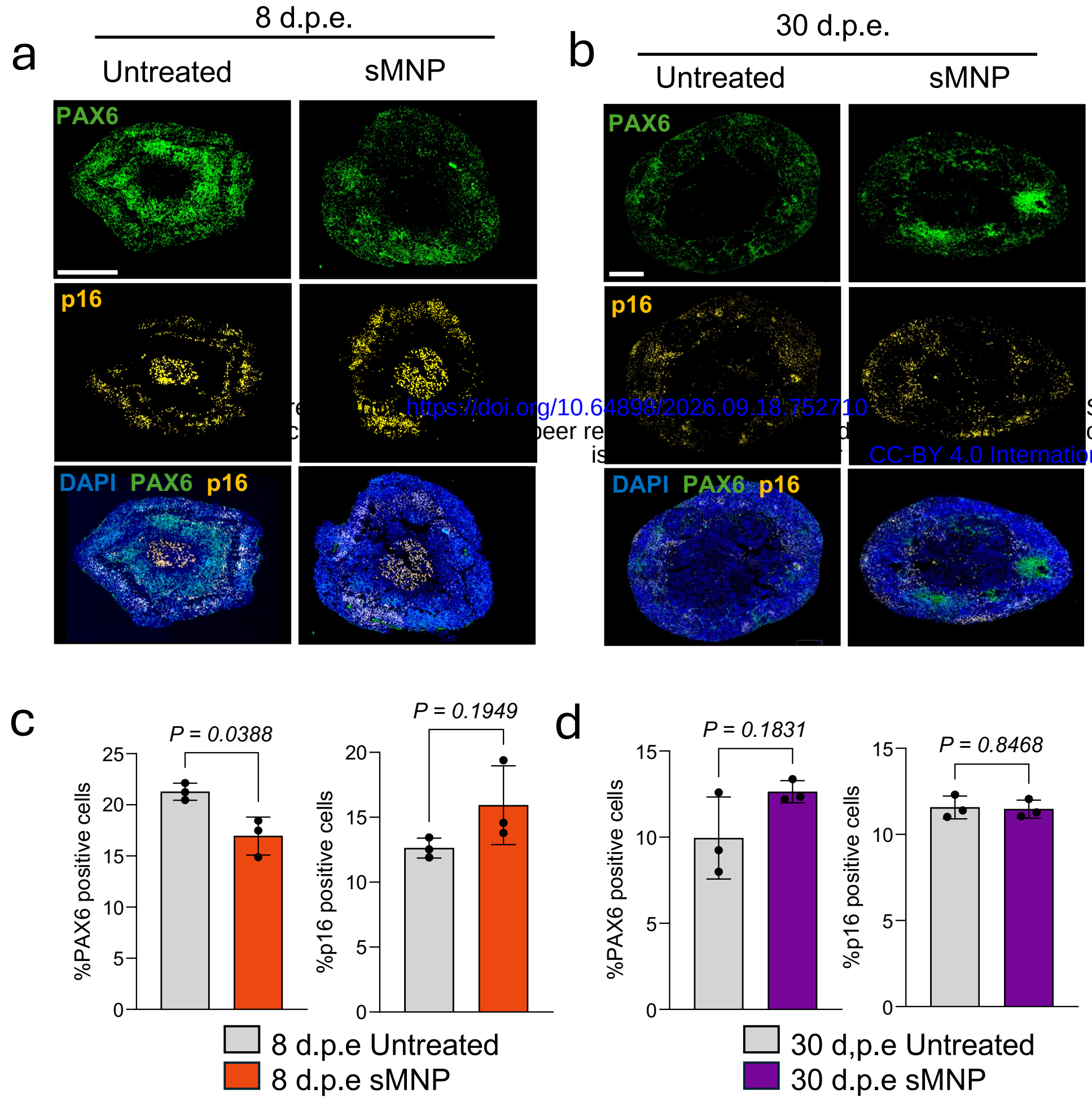

https://doi.org/10.64898/2026.09.18.752710 September 24, 2026. The copyright holder for this
bioRxiv a license to display the preprint in perpetuity
CC-BY 4.0 International license.

Supplementary Figure 5

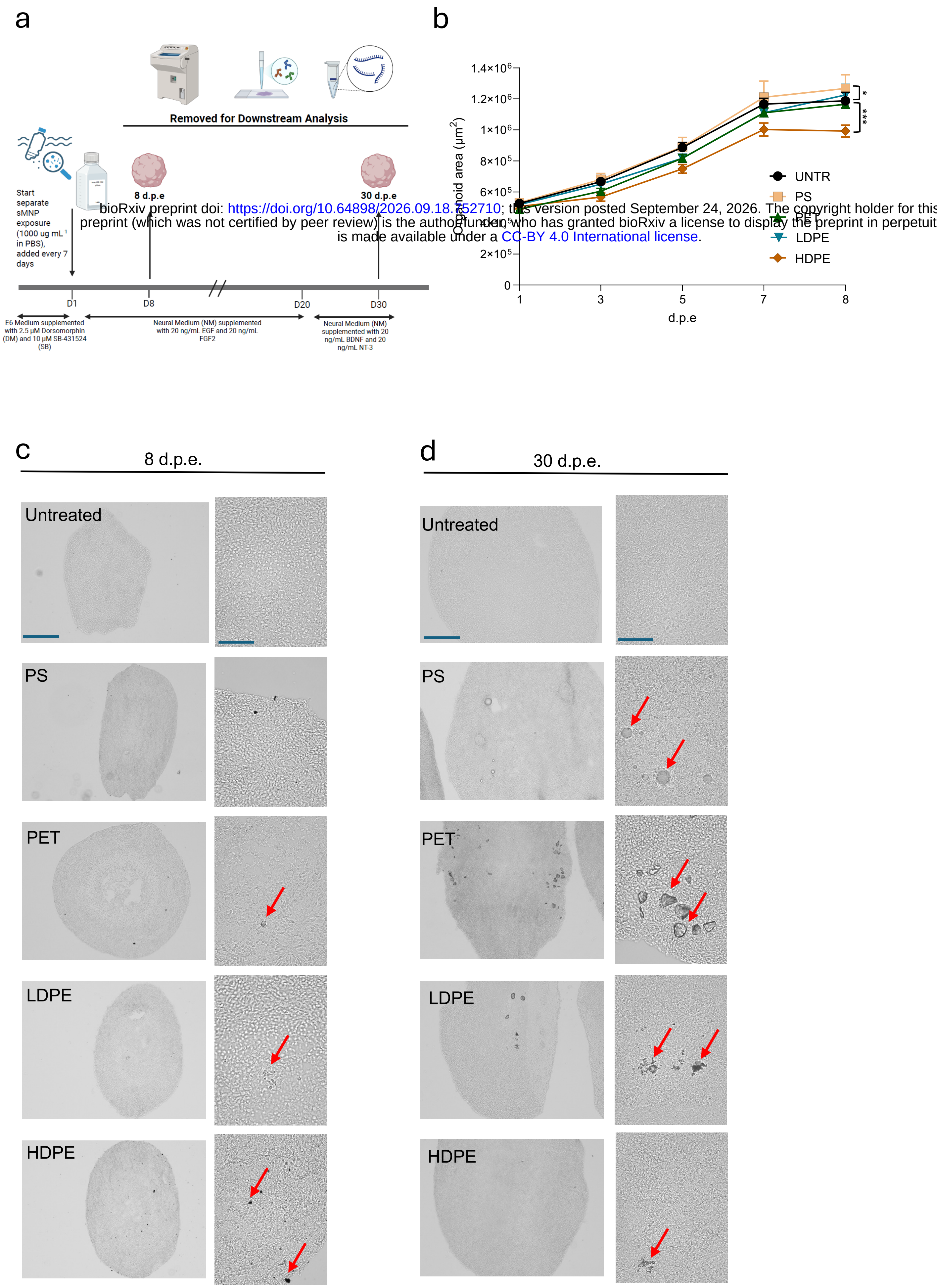

bioRxiv preprint doi: https://doi.org/10.64898/2026.09.18.752710; this version posted September 24, 2026. The copyright holder for this preprint (which was not certified by peer review) is the author/funder, who has granted bioRxiv a license to display the preprint in perpetuit
is made available under a CC-BY 4.0 International license.

Supplementary Figure 6

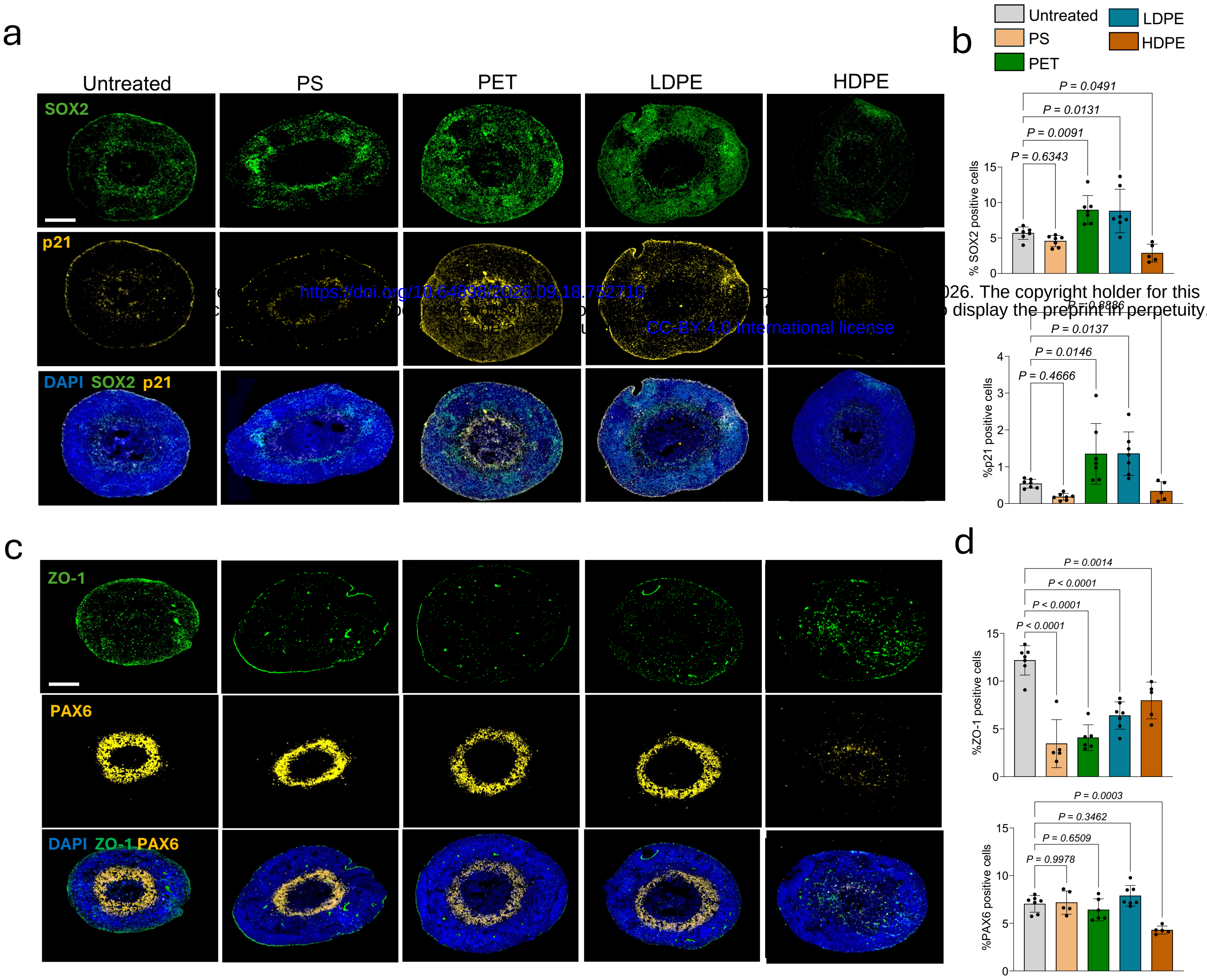

https://doi.org/10.64898/2026.09.18.752710
026. The copyright holder for this
display the preprint in perpetuity.
CC-BY 4.0 International license

Supplementary Figure 7

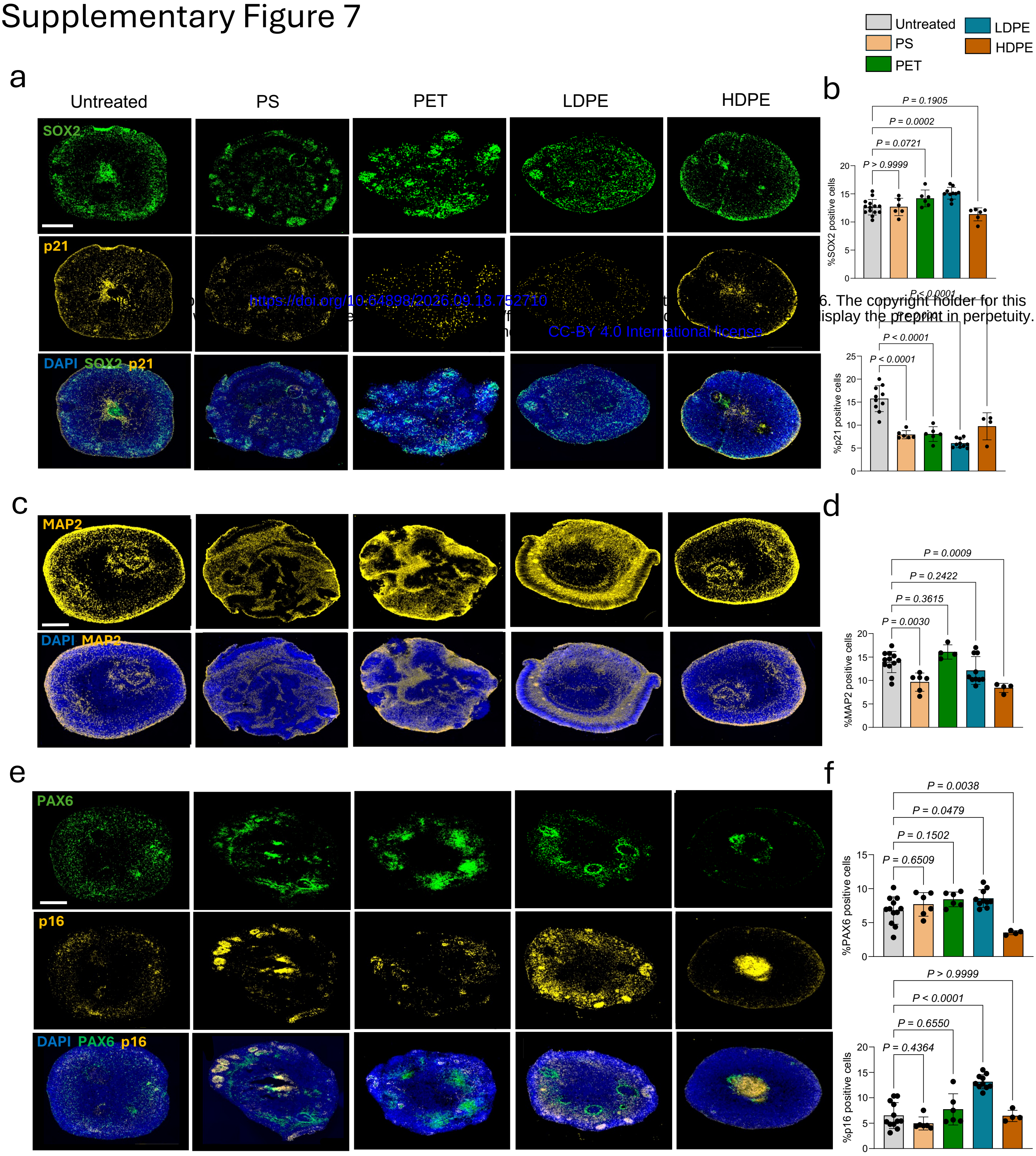

https://doi.org/10.64898/2025.09.18.752710 ... 6. The copyright holder for this ... isplay the preprint in perpetuity. ... CC-BY 4.0 International license

Supplementary Figure 8

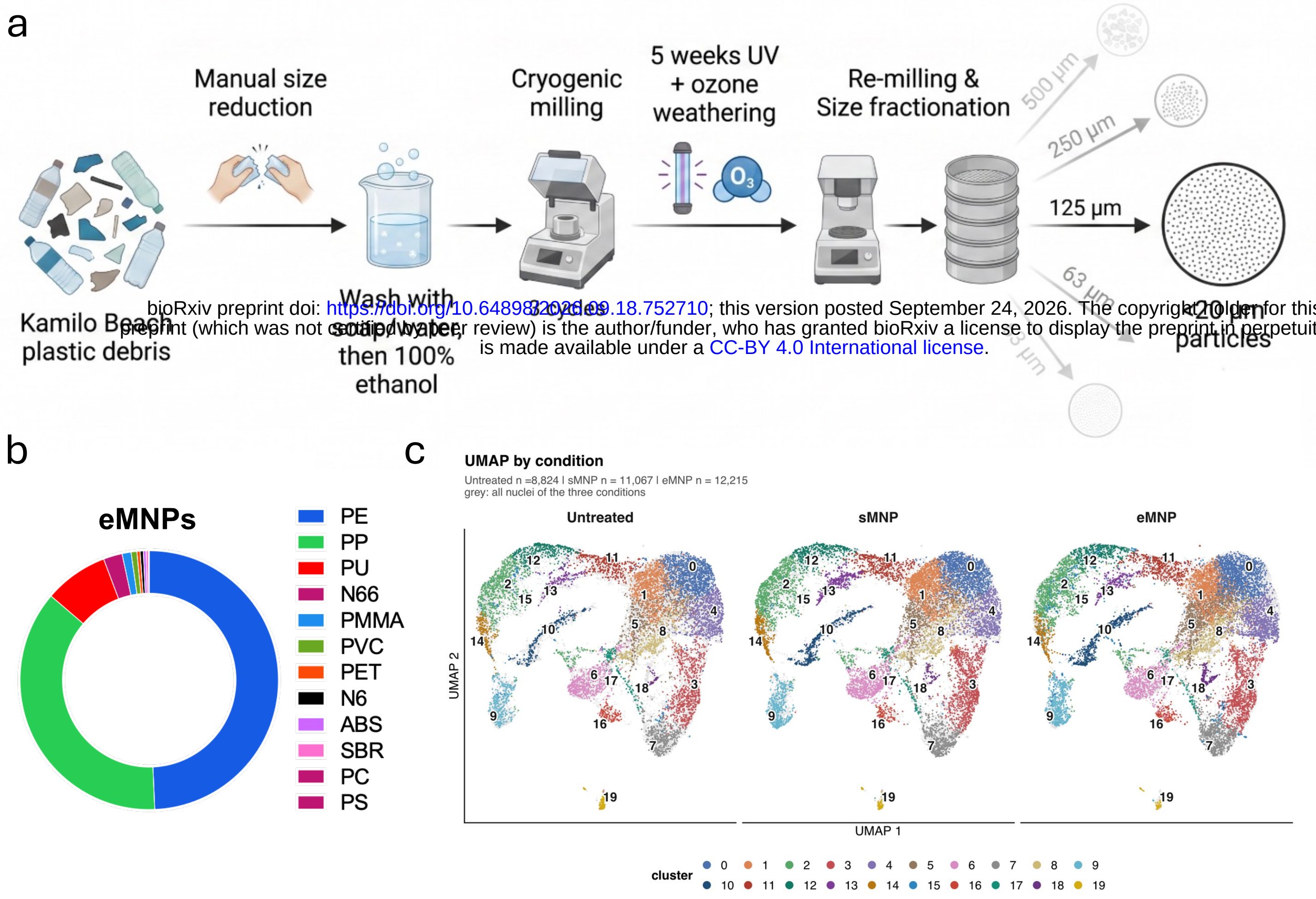

bioRxiv preprint doi: https://doi.org/10.64898/2026.09.18.752710; this version posted September 24, 2026. The copyright holder for this preprint (which was not certified by peer review) is the author/funder, who has granted bioRxiv a license to display the preprint in perpetuity. It is made available under a CC-BY 4.0 International license.

Supplementary Figure 9

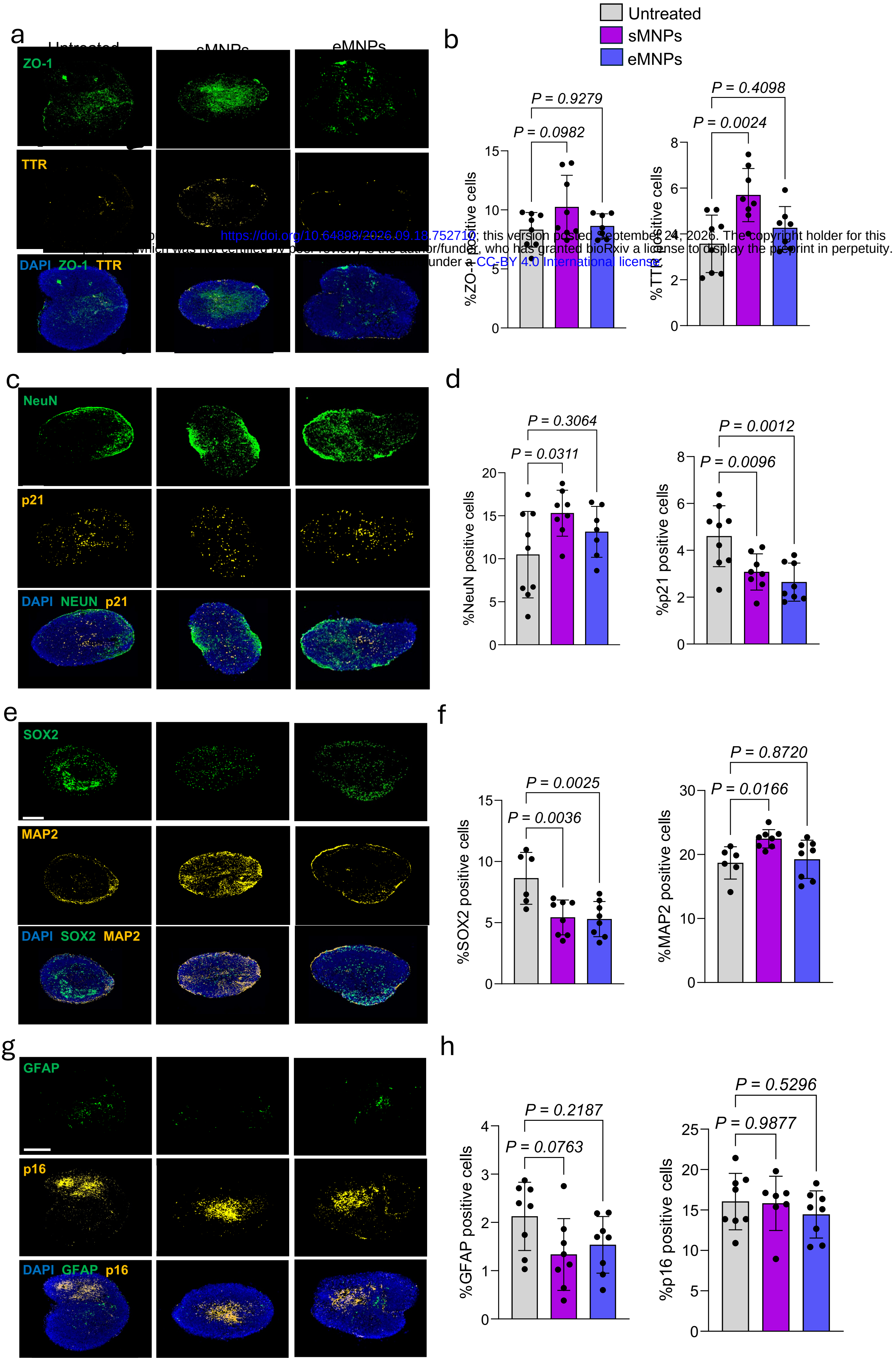

https://doi.org/10.64898/2026.09.18.752716; this version posted September 24, 2026. The copyright holder for this
(which was not certified by peer review) is the author/funder, who has granted bioRxiv a license to display the preprint in perpetuity.
under a CC-BY 4.0 International license.

Supplementary Figure 10

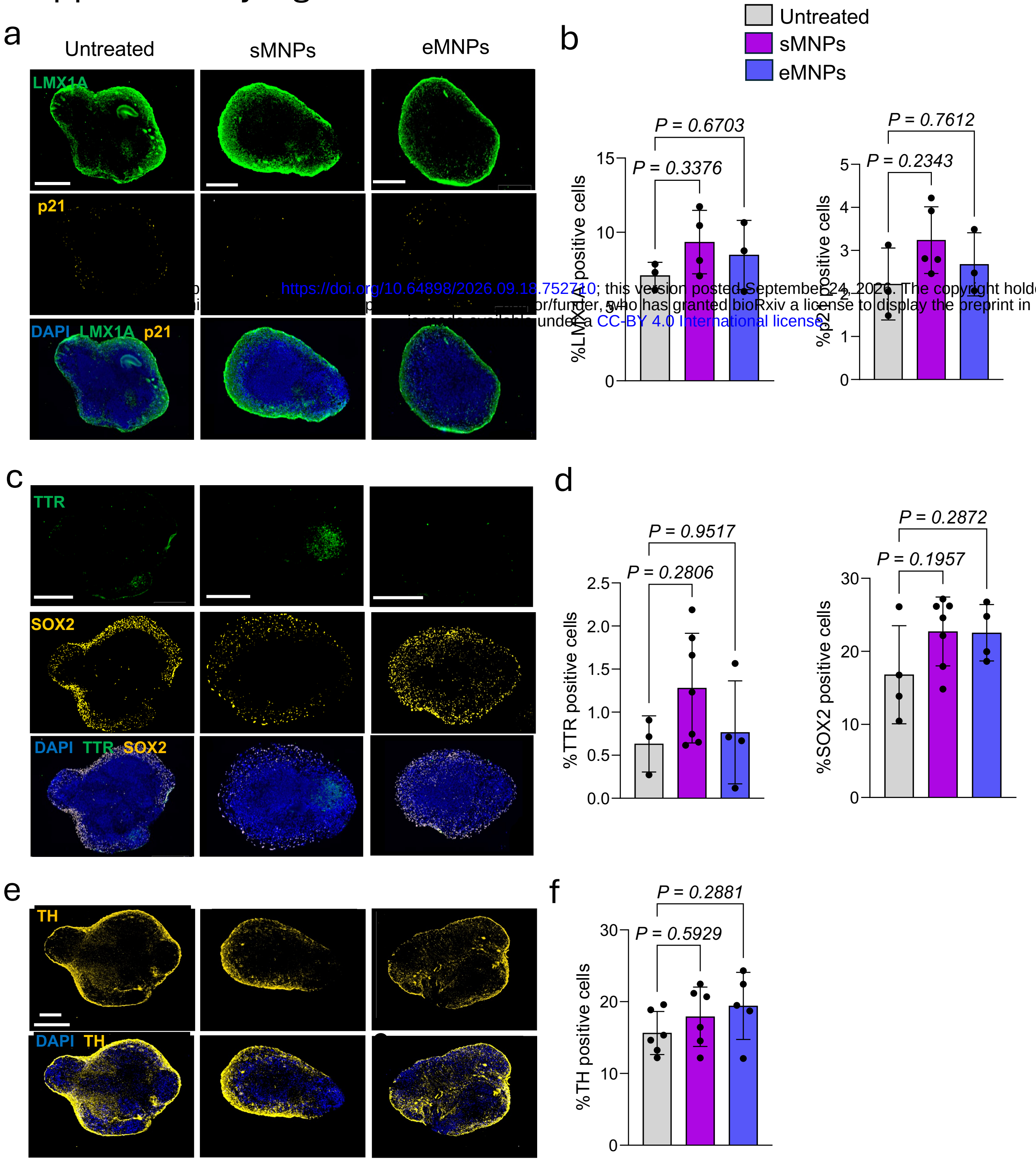

https://doi.org/10.64898/2026.09.18.752710; this version posted September 24, 2026. The copyright holder for this
or/funder, who has granted bioRxiv a license to display the preprint in perpetuity.
is made available under a CC-BY 4.0 International license.

Supplementary Figure 11

a

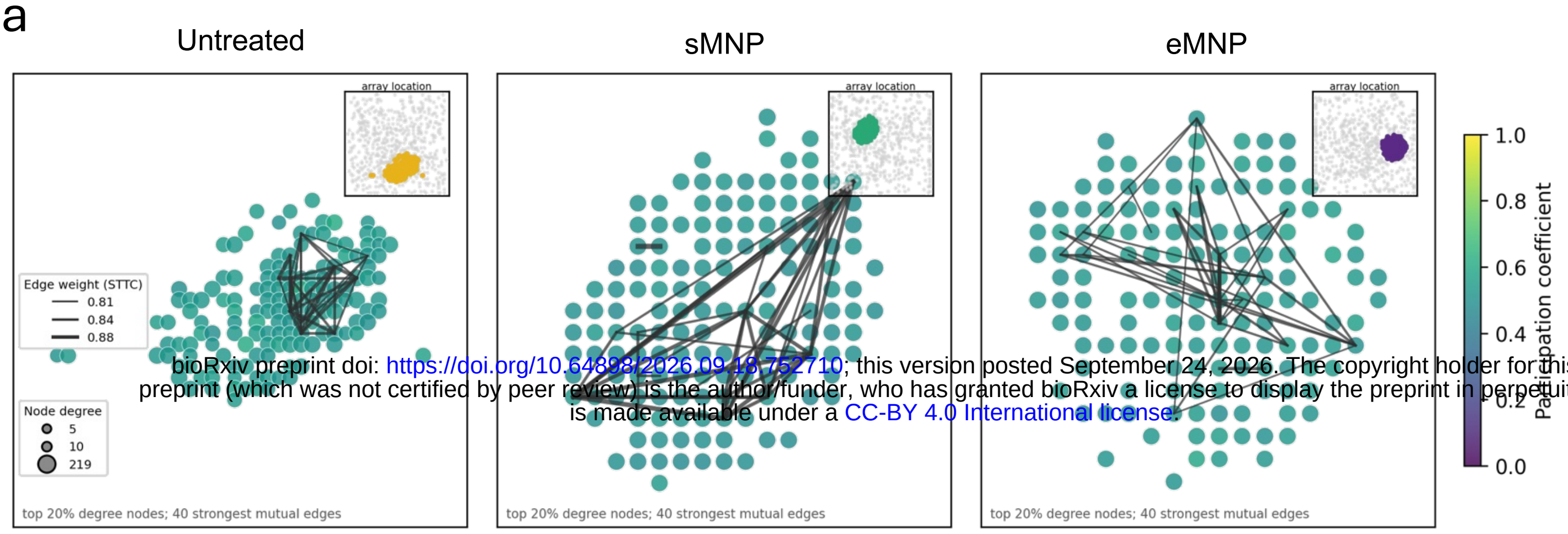

bioRxiv preprint doi: https://doi.org/10.64898/2026.09.18.752710; this version posted September 24, 2026. The copyright holder for this preprint (which was not certified by peer review) is the author/funder, who has granted bioRxiv a license to display the preprint in perpetuity. It is made available under a CC-BY 4.0 International license.
